# Metastasis of pulmonary adenocarcinoma to right occipital parafalcine meningioma: A case report and literature review

**DOI:** 10.1097/MD.0000000000023028

**Published:** 2020-10-30

**Authors:** Tianhao Hu, Run Wang, Yifu Song, Juanhan Yu, Zongze Guo, Sheng Han

**Affiliations:** a Department of Neurosurgery, The First Hospital of China Medical University; b Department of Pathology, China Medical University, Shenyang, China.

**Keywords:** lung carcinoma, meningioma, tumor-to-tumor metastasis

## Abstract

**Rationale:**

Tumor-to-tumor metastasis is a rare clinical phenomenon. Although meningioma is the most common intracranial recipient of cancer metastasis, only a few cases have been reported. We present a case of metastasis of lung adenocarcinoma into intracranial meningioma and review the published literature.

**Patient concerns:**

A 70-year-old woman was admitted to our hospital for a 1-month history of headache and pain in her lower extremities.

**Diagnosis:**

Brain and lumbar vertebral magnetic resonance imaging showed an intracranial space-occupying lesion in the right occipital region and spinal canal stenosis. Pulmonary computed tomography showed an irregular mass in the right upper lobe of the lung. The postoperative histological examination demonstrated adenocarcinoma metastasis to meningioma.

**Intervention:**

The patient underwent right occipital craniotomy for tumor removal and lumbar spinal canal decompression.

**Outcomes:**

There were no initial abnormal conditions after the operation. However, the patient died suddenly 7 days after surgery.

**Lessons:**

Tumor-to-meningioma metastasis is a rare but important phenomenon. According to previous reports, it is associated with rapid onset of symptoms and a poor prognosis. Histological examination is of great importance in diagnosis. The history and process of malignant carcinoma should be closely monitored.

## 1 Introduction

Metastasis from one tumor to another is known as tumor-to-tumor metastasis (TTM), which is a rare phenomenon.^[1]^ Meningioma, which constitutes 20% of intracranial tumors, is the most common intracranial recipient of systemic metastases.^[2]^ Breast and lung carcinomas are the most common origins of TTM.^[2,3]^ Because there have been few reported cases of metastasis to meningioma, the clinical characteristics of such patients are still unclear. According to previous reports, patients suffering from TTM have an extremely poor prognosis. Therefore, the accumulation of such cases is clinically relevant. Here, we report a case of lung adenocarcinoma metastasizing to meningioma.

## 2 Case report

This study was approved by the institutional review board at The First Hospital of China Medical University. Written consent was obtained from the family of the patient for publication of this case report and any accompanying images.

**Figure 1:**
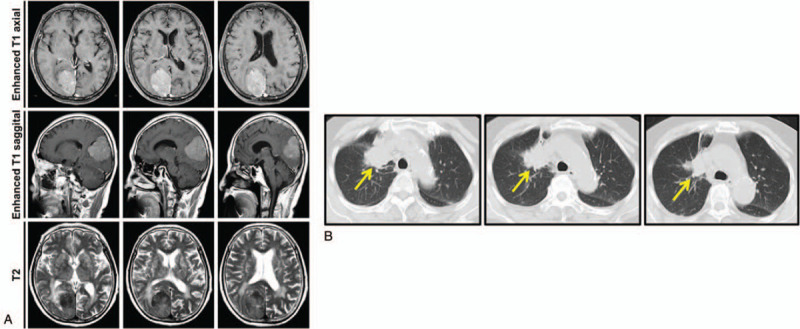
Imaging examinations of the brain and lungs before surgery. A, Preoperative contrast-enhanced MRI examination of the brain reveals a well-circumscribed mass (4.5 × 3.6 × 4.7 cm) in the right occipital parafalcine region. B, CT scan of the chest shows a 3.6 × 3.3 cm mass in the right upper lobe.

A 70-year-old woman was admitted to our institution with headache and pain in her lower extremities for 1 month, with the left side being more severe. Brain and lumbar vertebral magnetic resonance imaging (MRI) at the local hospital suggested an intracranial space-occupying lesion and spinal canal stenosis. Recently, she had suffered from pain in her waist and both hips and experienced laborious defecation. The patient had no history of smoking or drinking. She had grade 1 hypertension but no diabetes. We performed brain contrast-enhanced MRI and lumbar vertebral (L1–S1) 3-dimensional computed tomography (3D-CT). Brain MRI showed a well-circumscribed mass (4.5 × 3.6 × 4.7 cm) that had isointense signals on T1-weighted images and isointense signals with heterogeneity on T2-weighted image in the right occipital parafalcine region (Fig. 1A). Lumbar vertebral 3D-CT showed L3–S1 intervertebral disc bulge, ligamentum flavum thickening, and spinal canal stenosis. Lung CT showed an irregular mass in the upper lobe of the right lung (3.6 × 3.3 cm), bone destruction in the bilateral ribs, inflammation in the lower field of both lungs, and pleural effusion, which suggested a malignant lesion derived from the lung (Fig. 1B). Comprehensive analysis of pulmonary function showed mixed ventilation dysfunction, small airway dysfunction, and a ventilation reserve of 89%. Following the advice of a respiratory physician, the patient underwent atomization inhalation treatment with ipratropium bromide aerosol, budesonide suspension, and ambroxol hydrochloride for inhalation during the perioperative period. According to the imaging examination, the preoperative diagnosis of the patient was lung cancer, lumbar spinal stenosis, and right occipital meningioma or metastatic tumor. Although lung biopsy was recommended, the patient refused.

**Figure 2:**
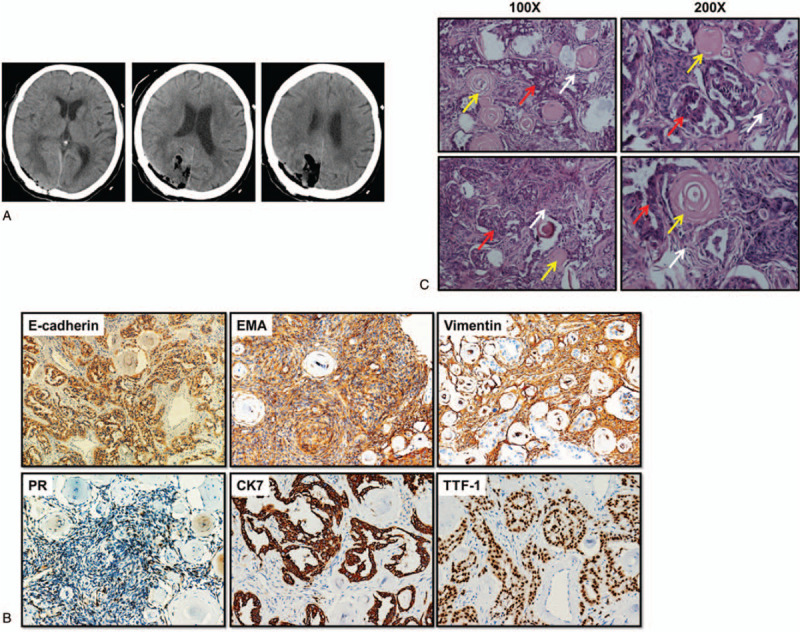
Postoperative CT reexamination and histopathological examination. A, Postoperative CT scan of brain shows that the tumor was totally removed. B, Immunohistochemistry showing the expression of E-cadherin, EMA, vimentin, PR, CK7, and TTF-1. C, Histopathological examination of the right occipital space occupied by the brain lesion was proved to be adenocarcinoma metastasizing to meningioma along with the presence of meningioma cells (white arrow), psammoma bodies (yellow arrow), and adenocarcinoma cells (red arrow) by hematoxylin and eosin (H&E) staining.

Subsequently, the patient underwent right occipital craniotomy for tumor removal (Simpson grade II resection) and lumbar spinal canal decompression. Postoperative brain CT revealed normal postoperative changes, and the tumor was totally removed (Fig. 2A). There were no initial abnormal conditions after the operation. However, the patient died suddenly of a cardiopulmonary accident 7 days after surgery. Due to the rapid deterioration of the patient, treatment for the lung lesion was not performed.

Immunohistochemically, the brain tumor stained positive for epithelial membrane antigen (EMA), progesterone receptor (PR), vimentin, and E-cadherin (Fig. 2B) and negative for glial fibrillary acidic protein (GFAP), S-100, p53, and oligodendrocyte transcription factor 2 (Olig2), which was consistent with WHO grade I meningioma. The focus within the meningioma stained positive for cytokeratin 7 (CK7) and thyroid transcription factor 1 (TTF-1; Fig. 2B) and negative for CK5/6, p63, CD56, and synaptophysin. TTF-1 and CK7 are markers expressed in adenocarcinoma of lung. Histologically, the brain tumor was psammomatous meningioma characterized by including numerous psammoma bodies (Fig. 2C, yellow arrow). Furthermore, there were hyperchromatic nuclei and prominent nucleoli cancer cells (Fig. 2C, red arrow) among meningioma cells (Fig. 2C, white arrow), which was consistent with metastatic carcinoma. The adenocarcinoma cells showed dense papillary hyperplasia with nuclear atypia (Fig. 2C, red arrow). Therefore, histopathological examination demonstrated adenocarcinoma metastasis to meningioma (Fig. 2C). Immunohistochemical and histopathological examinations were performed and reported by the Department of Pathology at China Medical University.

## 3 Discussion

TTM is a rare and well-recognized phenomenon.^[4,5]^ The most common malignant recipient tumor is renal cell carcinoma.^[1,2]^ Meningiomas are the most common benign tumors to harbor systemic metastases,^[6]^ but tumor-to-meningioma metastasis (TMM) has rarely been reported since the first case reported by Fried in 1930.^[7–9]^ To the best of our knowledge, there are fewer than 30 reports of lung carcinoma metastasis to meningioma.^[3–5,7,10–34]^ The epidemiology of TMM is still unknown. From January 2011 to January 2019, there were 2922 consecutive patients diagnosed with meningioma and 540 consecutive patients diagnosed with intracranial metastatic tumor at the Department of Neurosurgery at The First Hospital of China Medical University. There was only 1 TMM patient, accounting for 0.03% of meningioma and 0.19% of intracranial metastasis cases.

For diagnosis of TTM, Campbell et al proposed the following criteria: at least 2 primary tumors must exist, the metastatic focus must show established growth inside the host tumor and not be of contiguous growth, and the host tumor must be a true neoplasm and cannot be a lymph node involved in leukemia or lymphoma.^[1,35]^ Our case fulfilled the inclusion criteria for TTM established by Campbell et al. Previous studies presented different hypotheses related to the reasons why meningioma is the most common intracranial host in TTM. Meningiomas can provide an accessible and favorable environment for growth to receive metastases^[19]^ because they are highly vascular tumors^[36]^ and exhibit slow growth and an indolent nature.^[19,37]^ Furthermore, their high collagen and lipid content may provide a “fertile soil” for the seeding of malignant cells.^[6,19,20,38]^ Some researchers have suggested that cell–cell adhesion molecules, such as E-cadherin,^[39,40]^ may play a role in TMM.^[2,26,38,39]^ E-cadherin expression is downregulated when carcinoma cells escape from the primary tumor.^[41]^ Metastatic cells resume E-cadherin expression upon seeding their destination.^[14]^ It has been demonstrated that meningiomas highly express E-cadherin.^[40,42]^ Moreover, meningiomas harboring metastases are more likely to express E-cadherin than meningiomas in general.^[6]^ Therefore, the above evidence reveals that E-cadherin may play a role in TMM. Consistent with previous reports, in the present case, the tumor also exhibited high expression of E-cadherin, as demonstrated by immunohistochemistry (Fig. 2B). However, the relationship and underlying mechanism between E-cadherin and TMM requires further research. Psammoma bodies are concentric whorl calcification structures that exist in 45% of meningiomas.^[43]^ The possible protective role of psammoma bodies in the spread of TMM has been discussed in previous reports,^[11,22]^ and the meningioma in our case was rich in psammoma bodies.

We summarize the published lung carcinoma TMM cases in Table 1  and immunohistochemical results in Table 2. The mean age of patients was 65.03 years (range, 39–91 years), and there were 15 women and 14 men among the published cases (female:male = 1.07:1). According to the available immunohistochemical results of published cases, the meningioma components were often positive for EMA, PR, and vimentin (except for 1 case of secretory meningioma), and the pulmonary carcinoma components were frequently positive for TTF-1 and CK7. All the cases were supratentorial lesions. Except for 1 case of atypical meningioma, the others were benign meningiomas. The most common type of lung carcinoma was adenocarcinoma (69.0%). Most of them were discovered by chance at surgery or autopsy and had the feature of a previously existing malignant tumor. However, cases of TMM of occult lung malignant tumors have rarely been reported.^[5,23,27,31]^ In our case, there were no preoperative symptoms of lung carcinoma except for an irregular mass on imaging examinations. Many authors have considered it difficult for clinicians to provide a specific preoperative diagnosis of TMM by imaging examination.^[2,27,28]^ There are no conclusive MRI features.^[25,27,28]^ Danisman Specialist et al suggested that perfusion MRI is an advantageous preoperative proposal for differential diagnosis of meningioma.^[33]^

**Table 1:**
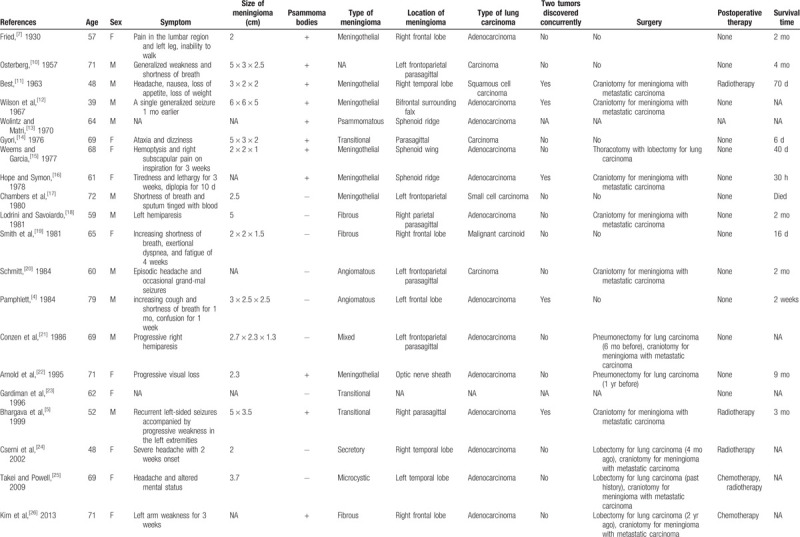
Summary of cases of lung carcinoma metastasis to intracranial meningioma.

**Table 1 (Continued):**
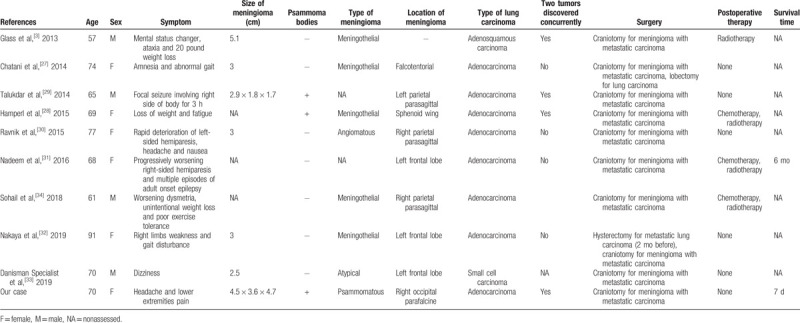
Summary of cases of lung carcinoma metastasis to intracranial meningioma.

**Table 2:**
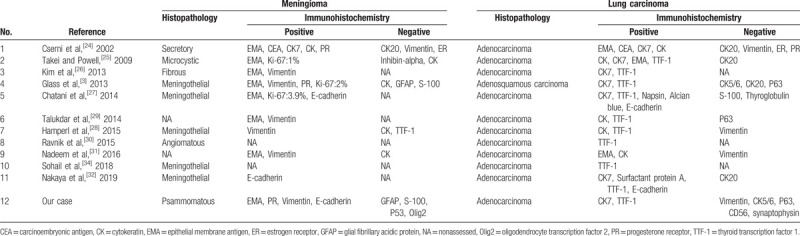
Immunohistochemical results of published lung carcinoma metastasis to meningioma.

Lung carcinoma is known to commonly metastasize to the brain, with 10% to 36% of all lung carcinomas developing brain metastasis.^[44]^ The average survival of patients with brain metastasis is less than 6 months.^[45,46]^ Whole-brain radiotherapy is the main treatment for brain metastases, but it is limited by long-term side effects.^[47]^ A combination of stereotactic and whole-brain radiotherapy for brain metastases can significantly improve local control.^[48]^ In the previous reports, 7 patients (24.1%) underwent lung lobectomy. Nineteen patients (65.5%) underwent craniotomy. In patients without early positive intervention for malignant carcinoma, the course of the disease often deteriorated quickly, and they had poor prognosis. For intracranial lesions of TMM, surgical indications are usually space-occupying effects and central nervous system symptoms. In the present case, the intracranial tumor had a maximum diameter of 4.7 cm. Based on the preoperative imaging examination, it was considered to be meningioma, although metastatic tumor could not be excluded because of the irregular mass in the lung. Therefore, surgical resection was performed. Owing to incomplete follow-up information in previous case reports and the rarity of the disease, we are unable to summarize the specific survival period. According to the data available to us, the shortest postoperative survival time is 30 hours, and the longest is 9 months. The patient in our case suddenly developed dyspnea and cardiac arrest at 7 days postoperatively. Although rescue was performed, her condition continued to deteriorate. Our patient died shortly after surgery, and since autopsy was not performed, we were unable to determine the exact cause of death. However, we should carefully assess the systemic status of patients and surgical indications, because coexistent malignancy and TMM might significantly affect patients’ physical function. Therefore, aggressive surgical treatment should be carefully considered and advised. Due to the poor prognosis, it is necessary to develop an optimal management method for these superimposed malignancies.

## 4 Conclusion

TMM is a rare but important phenomenon. The precise mechanisms of this unique event remain undefined, and most patients have an extremely poor prognosis. Histological examination is the only diagnostic approach. The history and clinical process of TMM should be closely monitored.

## Author contributions

**Conceptualization:** Tianhao Hu.

**Funding acquisition:** Sheng Han.

**Investigation:** Juanhan Yu.

**Methodology:** Sheng Han.

**Resources:** Run Wang, Yifu Song, Zongze Guo.

**Writing – original draft:** Tianhao Hu.

**Writing – review & editing:** Sheng Han.

## References

[1] Campbell L Gilbert E Chamberlain C Metastases of cancer to cancer. Cancer 1968;22:635–43. 5673241 10.1002/1097-0142(196809)22:3<635::aid-cncr2820220320>3.0.co;2-o

[2] Sayegh ET Burch EA Henderson GA Tumor-to-tumor metastasis: breast carcinoma to meningioma. J Clin Neurosci 2015;22:268–74. 25150768 10.1016/j.jocn.2014.07.002

[3] Glass R Hukku S Gershenhorn B Metastasis of lung adenosquamous carcinoma to meningioma: case report with literature review. Int J Clin Exp Pathol 2013;6:2625–30. 24228131 PMC3816838

[4] Pamphlett R Carcinoma metastasis to meningioma. J Neurol Neurosurg Psychiatry 1984;47:561–3. 6330308 10.1136/jnnp.47.5.561 PMC1027839

[5] Bhargava P McGrail K Manz H Lung carcinoma presenting as metastasis to intracranial meningioma: case report and review of the literature. Am J Clin Oncol 1999;22:199–202. 10199462 10.1097/00000421-199904000-00022

[6] Aghi M Kiehl TR Brisman JL Breast adenocarcinoma metastatic to epidural cervical spine meningioma: case report and review of the literature. J Neurooncol 2005;75:149–55. 16132512 10.1007/s11060-005-1408-4

[7] Fried B Metastatic inoculation of a meningioma by cancer cells from a bronchogenic carcinoma. Am J Pathol 1930;6:47–52. 19969886 PMC2007275

[8] Erdogan H Aydin M Tasdemiroglu E Tumor-to-tumor metastasis of the central nervous system. Turk Neurosurg 2014;24:151–62. 24831354 10.5137/1019-5149.JTN.8317-13.1

[9] Neville IS Solla DF Oliveira AM Suspected tumor-to-meningioma metastasis: a case report. Oncol Lett 2017;13:1529–34. 28454286 10.3892/ol.2017.5655 PMC5403379

[10] Osterberg D Metastases of carcinoma to meningioma. J Neurosurg 1957;14:337–43. 13429401 10.3171/jns.1957.14.3.0337

[11] Best P Metastatic carcinoma in a meningioma: report of a case. J Neurosurg 1963;20:892–4. 14186085 10.3171/jns.1963.20.10.0892

[12] Wilson C Jenevein E Bryant L Carcinoma of the lung metastatic to falx meningioma: case report. J Neurosurg 1967;27:161–5.

[13] Wolintz A Matri A Metastasis of carcinoma of lung to sphenoid ridge meningioma. N Y State J Med 1970;70:2592–8. 5272944

[14] Gyori E Metastatic carcinoma in meningioma. South Med J 1976;69:514–7. 1265521 10.1097/00007611-197604000-00042

[15] Weems T Garcia J Intracranial meningioma containing metastatic foci. South Med J 1977;70:503–5. 191937 10.1097/00007611-197704000-00044

[16] Hope D Symon L Metastasis of carcinoma to meningioma. Acta Neurochir 1978;40:307–13. 676808 10.1007/BF01774755

[17] Chambers P Davis R Blanding MJ Metastases to primary intracranial meningioma and neurilemomas. Arch Pathol Lab Med 1980;104:350–4. 6893121

[18] Lodrini S Savoiardo M Metastases of carcinoma to intracranial meningioma: report of two cases and review of the literature. Cancer 1981;48:2668–73. 7306923 10.1002/1097-0142(19811215)48:12<2668::aid-cncr2820481219>3.0.co;2-n

[19] Smith T Schoene W Wang S Malignant carcinoid tumor metastatic to a meningioma. Cancer 1981;47:1872–7. 7226082 10.1002/1097-0142(19810401)47:7<1872::aid-cncr2820470726>3.0.co;2-2

[20] Schmitt H Metastases of malignant neoplasms to intracranial tumors: the “tumor-in-a tumor” phenomenon. Virchows Arch 1984;405:155–60. 10.1007/BF00694933 6438898

[21] Conzen M Sollmann H Schnabel R Metastasis of lung carcinoma to intracranial meningioma: case report and review of literature. Neurochirurgia (Stuttg) 1986;29:206–9. 3785498 10.1055/s-2008-1054162

[22] Arnold A Hepler R Badr M Metastasis of adenocarcinoma of the lung to optic nerve sheath meningioma. Arch Ophthalmol 1995;113:346–51. 7887848 10.1001/archopht.1995.01100030102029

[23] Gardiman M Altavilla G Marchioro L Metastasis to intracranial meningioma as first clinical manifestation of occult primary lung carcinoma. Tumori 1996;82:256–8. 8693606

[24] Cserni G Bori R Huszka E Metastasis of pulmonary adenocarcinoma in right Sylvian secretory meningioma. Br J Neurosurg 2002;16:66–8. 11926470 10.1080/026886902753512646

[25] Takei H Powell SZ Tumor-to-tumor metastasis to the central nervous system. Neuropathology 2009;29:303–8. 18647266 10.1111/j.1440-1789.2008.00952.x

[26] Kim KH Hong EK Lee SH Non small cell carcinoma metastasis to meningioma. J Korean Neurosurg Soc 2013;53:43–5. 23441002 10.3340/jkns.2013.53.1.43 PMC3579081

[27] Chatani M Nakagawa I Yamada S Intracranial meningioma as initial clinical manifestation of occult lung carcinoma: case report. Neurol Med Chir (Tokyo) 2014;54:670–2. 24305014 10.2176/nmc.cr.2013-0061 PMC4533486

[28] Hamperl M Goehre F Schwan S Tumor-to-tumor metastasis – bronchial carcinoma in meningioma. Clin Neuropathol 2015;34:302–6. 26073490 10.5414/NP300856

[29] Talukdar A Khanra D Mukhopadhay S Tumor to tumor metastasis: adenocarcinoma of lung metastatic to meningioma. J Postgrad Med 2014;60:403–5. 25370552 10.4103/0022-3859.143974

[30] Ravnik J Ravnik M Bunc G Metastasis of an occult pulmonary carcinoma into meningioma: a case report. World J Surg Oncol 2015;13:292. 26438229 10.1186/s12957-015-0714-3 PMC4595197

[31] Nadeem M Assad S Nasir H Intrameningioma metastasis: clinical manifestation of occult primary lung carcinoma. Cureus 2016;8:e704. 27588225 10.7759/cureus.704 PMC4999154

[32] Nakaya M Ichimura S Kurebayashi Y Contiguous metastasis of pulmonary adenocarcinoma to meningioma. J Neurol Surg A Cent Eur Neurosurg 2019;80:127–30. 30321884 10.1055/s-0038-1669471

[33] Danisman Specialist MC Koplay M Paksoy Y Small cell lung carcinoma metastasis to atypical meningioma: the importance of perfusion MRI graphics in differential diagnosis. World Neurosurg 2019;124:410–3. 10.1016/j.wneu.2019.01.084 30703595

[34] Syed S Karambizi DI Baker A A comparative report on intracranial tumor-to-tumor metastasis and collision tumors. World Neurosurg 2018;116:454–63. e2. 29704691 10.1016/j.wneu.2018.04.109

[35] Pham J Kim R Nguyen A Intracranial meningioma with carcinoma tumor-to-tumor metastasis: two case reports. CNS Oncol 2018;7:CNS09. 29698064 10.2217/cns-2017-0022 PMC5977278

[36] Caroli E Salvati M Giangaspero F Intrameningioma metastasis as first clinical manifestation of occult primary breast carcinoma. Neurosurg Rev 2006;29:49–54. 16133455 10.1007/s10143-005-0395-4

[37] Zeidman LA Ankenbrandt WJ Du H Growth rate of non-operated meningiomas. J Neurol 2008;255:891–5. 18350353 10.1007/s00415-008-0801-2

[38] Watanabe T Fujisawa H Hasegawa M Metastasis of breast cancer to intracranial meningioma. Am J Clin Oncol 2002;25:414–7. 12151976 10.1097/00000421-200208000-00019

[39] Lanotte M Benech F Panciani PP Systemic cancer metastasis in a meningioma: report of two cases and review of the literature. Clin Neurol Neurosurg 2009;111:87–93. 18930586 10.1016/j.clineuro.2008.07.011

[40] Shimada S Ishizawa K Hirose T Expression of E-cadherin and catenins in meningioma: ubiquitous expression and its irrelevance to malignancy. Pathol Int 2005;55:1–7. 15660696 10.1111/j.1440-1827.2005.01786.x

[41] Arnold S Young A Munn R Expression of p53, bcl-2, E-cadherin, matrix metalloproteinase-9, and tissue inhibitor of metalloproteinases-1 in paired primary tumors and brain metastasis. Clin Cancer Res 1999;5:4028–33. 10632335

[42] Figarella-Branger D Pellissier J Bouillot P Expression of neural cell-adhesion molecule isoforms and epithelial cadherin adhesion molecules in 47 human meningiomas: correlation with clinical and morphological data. Mod Pathol 1994;7:752–61. 7824509

[43] Das DK Psammoma body: a product of dystrophic calcification or of a biologically active process that aims at limiting the growth and spread of tumor? Diagn Cytopathol 2009;37:534–41. 19373908 10.1002/dc.21081

[44] Villano JL Durbin EB Normandeau C Incidence of brain metastasis at initial presentation of lung cancer. Neuro Oncol 2015;17:122–8. 24891450 10.1093/neuonc/nou099 PMC4483041

[45] Fox BD Cheung VJ Patel AJ Epidemiology of metastatic brain tumors. Neurosurg Clin N Am 2011;22:1–6. 21109143 10.1016/j.nec.2010.08.007

[46] Stelzer KJ Epidemiology and prognosis of brain metastases. Surg Neurol Int 2013;4:S192–202. 23717790 10.4103/2152-7806.111296 PMC3656565

[47] Lim S Lee J Lee M A randomized phase III trial of stereotactic radiosurgery (SRS) versus observation for patients with asymptomatic cerebral oligo-metastases in non-small-cell lung cancer. Ann Oncol 2014;26:762–8. 25538174 10.1093/annonc/mdu584

[48] Khan M Lin J Liao G Whole brain radiation therapy plus stereotactic radiosurgery in the treatment of brain metastases leading to improved survival in patients with favorable prognostic factors. Front Oncol 2019;9:205. 30984624 10.3389/fonc.2019.00205 PMC6449627

